# Extended mobility scale (AMEXO) for assessing mobilization and setting goals after gastrointestinal and oncological surgery: a before-after study

**DOI:** 10.1186/s12893-021-01445-3

**Published:** 2022-02-02

**Authors:** José L. Boerrigter, Sven J. G. Geelen, Mark I. van Berge Henegouwen, Willem A. Bemelman, Susan van Dieren, Janneke M. de Man-van Ginkel, Marike van der Schaaf, Anne M. Eskes, Marc G. Besselink

**Affiliations:** 1 Department of Surgery, Amsterdam UMC, University of Amsterdam, Cancer Center Amsterdam, Amsterdam, The Netherlands; 2 Nursing Sciences, Program in Clinical Health Sciences, University Medical Center Utrecht, University Utrecht, Utrecht, The Netherlands; 3 Department of Rehabilitation Medicine, Amsterdam Movement Sciences, University of Amsterdam, Amsterdam UMC, Meibergdreef 9, 1105 AZ Amsterdam, The Netherlands; 4 Department of Nursing Science, Julius Center for Health Sciences and Primary Care, University Medical Center Utrecht, University Utrecht, Utrecht, The Netherlands; 5 Center of Expertise Urban Vitality, Faculty of Health, Amsterdam University of Applied Sciences, Amsterdam, The Netherlands; 6 Menzies Health Institute Queensland and School of Nursing and Midwifery, Griffith University, Gold Coast, Australia

**Keywords:** Early ambulation [MESH], Mobility limitation [MESH], Postoperative period [MESH], Patient outcome assessment [MESH]

## Abstract

**Background:**

Early structured mobilization has become a key element of Enhanced Recovery After Surgery programs to improve patient outcomes and decrease length of hospital stay. With the intention to assess and improve early mobilization levels, the 8-point ordinal John Hopkins Highest Level of Mobility (JH-HLM) scale was implemented at two gastrointestinal and oncological surgery wards in the Netherlands. After the implementation, however, healthcare professionals perceived a ceiling effect in assessing mobilization after gastrointestinal and oncological surgery. This study aimed to quantify this perceived ceiling effect, and aimed to determine if extending the JH-HLM scale with four additional response categories into the AMsterdam UMC EXtension of the JOhn HOpkins Highest Level of mObility (AMEXO) scale reduced this ceiling effect.

**Methods:**

All patients who underwent gastrointestinal and oncological surgery and had a mobility score on the first postoperative day before (July–December 2018) or after (July–December 2019) extending the JH-HLM into the AMEXO scale were included. The primary outcome was the before-after difference in the percentage of ceiling effects on the first three postoperative days. Furthermore, the before-after changes and distributions in mobility scores were evaluated. Univariable and multivariable logistic regression analysis were used to assess these differences.

**Results:**

Overall, 373 patients were included (JH-HLM n = 135; AMEXO n = 238). On the first postoperative day, 61 (45.2%) patients scored the highest possible mobility score before extending the JH-HLM into the AMEXO as compared to 4 (1.7%) patients after (OR = 0.021, CI = 0.007–0.059, *p* < 0.001). During the first three postoperative days, 118 (87.4%) patients scored the highest possible mobility score before compared to 40 (16.8%) patients after (OR = 0.028, CI = 0.013–0.060, *p* < 0.001). A change in mobility was observed in 88 (65.2%) patients before as compared to 225 (94.5%) patients after (OR = 9.101, CI = 4.046–20.476, *p* < 0.001). Of these 225 patients, the four additional response categories were used in 165 (73.3%) patients.

**Conclusions:**

A substantial ceiling effect was present in assessing early mobilization in patients after gastrointestinal and oncological surgery using the JH-HLM. Extending the JH-HLM into the AMEXO scale decreased the ceiling effect significantly, making the tool more appropriate to assess early mobilization and set daily mobilization goals after gastrointestinal and oncological surgery.

**Supplementary Information:**

The online version contains supplementary material available at 10.1186/s12893-021-01445-3.

## Introduction

Annually, over 300 million patients undergo surgery worldwide [1]. Prolonged bed rest and reduced mobility after surgical procedures have been associated with increased risk of complications [2, 3]. Previous research showed that patients after abdominal oncological surgery stay in bed with a median of 19 h a day during the first three postoperative days and walk only six minutes a day [4].

In order to facilitate postoperative recovery, the enhanced recovery after surgery (ERAS) program has been well established worldwide [5–7]. The implementation of ERAS program had decreased hospital stay and reduced postoperative complications [8]. A key element of the ERAS program is early mobilization, which entails the incremental increase in activity ranging from passive range-of-motion exercises to active ambulation, depending on the physical capabilities of the patient, from the first day after surgery to reach predetermined targets using a standardized and structured approach [6, 7, 9, 10]. Previous research showed that multifaceted interventions aimed at creating a culture that made safe and early mobilization possible resulted in significant and sustained improvement of patient mobilization levels [11].

With the intention to create a culture of safe and early mobilization, the John Hopkins University Hospital developed and implemented the Activity Measure for Post-Acute Care (AM-PAC) “6-clicks” Basic Mobility Short Form to assess limitations in functional mobility (i.e., what the patient is capable of doing) and the John Hopkins Highest Level of Mobility scale (JH-HLM) to assess mobilization (i.e., what a patient has actually done), set mobilization goals and discuss mobilization success during inter-professional meetings [12–14]. Specifically, the JH-HLM is a validated 1-item ordinal scale ranging from lying passively in bed (score = 1) to walking ≥ 250 ft (score = 8) [12, 13]. Previous research showed that the JH-HLM has excellent test–retest reliability and inter-rater reliability for nurses and physical therapists [13]. To sustainably improve the mobilization levels in Dutch hospitalized patients, the JH-HLM scale was implemented in several Dutch hospitals.

After the implementation of the JH-HLM scale at our tertiary university medical center, however, healthcare professionals perceived a ceiling effect in patients after gastrointestinal and oncological surgery as they noticed that at least half of the patients scored the highest possible score on the first day postoperative. Floor and ceiling effects are considered to be present if respectively more than 15% of the patients achieved the lowest or highest possible score, limiting the usability, reliability and responsiveness of the tool [15]. In the case of the JH-HLM, this ceiling effect hampered the multidisciplinary team in adequately assessing mobilization after gastrointestinal and oncological surgery, setting mobilization goals and discussing mobilization success during inter-professional meetings.

Here, in our effort to provide the multidisciplinary team with a tool that can adequately be used after gastrointestinal and oncological surgery, we extended the JH-HLM scale by adding four additional response categories into the AMsterdam UMC EXtension of the JOhn HOpkins Highest Level of mObility (AMEXO) scale. The AMEXO scale was subsequently implemented in routine clinical practice. If the ceiling effects were reduced by extending the JH-HLM into the AMEXO scale, the multidisciplinary team may be better able to assess mobilization, set mobilization goals and discuss mobilization success in patients after gastrointestinal and oncological surgery. Therefore, the aim of this study was two-folded. First, to quantify the perceived ceiling effect of the JH-HLM scale when the multidisciplinary team used it to assess mobility in patients after gastrointestinal and oncological surgery. Second, to determine if extending the JH-HLM scale with four additional response categories into the AMEXO scale reduced this ceiling effect.

## Methods

### Study design

This is an uncontrolled before-after study performed at two surgical wards. First, the JH-HLM scale was implemented at both surgical wards between March and June 2018, after which the JH-HLM scale was used during routine clinical care from July 2018 until January 2019. Second, the JH-HLM was extended into the AMEXO scale where after the AMEXO scale was implemented in routine care between February 2019 and June 2019 to assess mobilization and set daily mobilization goals during routine care instead of the JH-HLM scale. Data were extracted from the electronic medical records in January 2019 (before extending the JH-HLM into the AMEXO scale) and January 2020 (after extending the JH-HLM into the AMEXO scale) to quantify and compare both tools when used to assess mobilization in gastrointestinal surgical patients.

This study has been conducted according to the principles of the Declaration of Helsinki. The Medical Ethical Review Committee of the Amsterdam UMC, location Academic Medical Center, assessed and approved this study (reference number W19_034 # 19.053). As the dataset was supplied by the medical center and included only de-identified (anonymous) data; the Medical Ethical Review Committee waived the need for individual informed consents. The study was reported following the Strengthening the Reporting of Observational Studies in Epidemiology (STROBE) guidelines for cohort studies [16].

### Study population

All adult patients who were admitted to one of the two surgical wards between July and December 2018 (before) or July and December 2019 (after extending the JH-HLM into the AMEXO scale), underwent gastrointestinal and oncological surgery and had a JH-HLM or AMEXO score on the first postoperative day were included. Moreover, every patient was only included once, meaning that all subsequent hospital admissions were excluded from the analysis.

### JH-HLM and AMEXO mobility scales

The JH-HLM scale is a 1-item ordinal scale with eight response categories and is used by healthcare professionals to assess mobilization, set mobilization goals and discuss mobilization success during inter-professional meetings [13]. Each category is numbered consecutively from 1 = lying passively in bed to 8 = walking approximately 250 ft or more [13]. Initially, the JH-HLM scale has been developed to assist healthcare professionals caring for hospitalized general medicine patients [12]; however, the JH-HLM scale has also been used more recently in hospitalized adults at acute care units[17], hospitalized geriatric patients[18], hospitalized adults at a neuroscience/brain rescue unit[19, 20], surgical unit[21] or intensive care unit[22]. Using a convenience sample of hospitalized adults, Hoyer et al. showed that the test–retest reliability values for physical therapists and nurses (Intraclass Correlation Coefficients 0.94 and 0.95, respectively) and interrater reliability values between physical therapists and nurses (Intraclass Correlation Coefficient 0.99) were excellent [13]. Furthermore, the Standard Error of Measurement was 0.2, the Minimal Detectable Change (MDC_95_) was 0.6, and evidence was provided that the JH-HLM measured constructs of the ICF domain ‘mobility’ [13, 23]. To ensure clarity and ease of use for patients and healthcare professionals in our hospital, 25 and 250 ft was rounded to 7.5 and 75 m, respectively, instead of 7.62 and 76.2.

At first, the JH-HLM was implemented at our hospital and we placed meter markings on the walls to facilitate healthcare professionals in estimating the achieved JH-HLM score and in setting mobilization goals together with hospitalized adult patients. The highest JH-HLM score (i.e., 8 = 250 ft) represents a functional household ambulation distance and is estimated as 4 metabolic equivalents [12]. In three team discussions in January 2019, a multidisciplinary team involving surgeons, physicians, nurses, physical therapists and researchers evaluated the distribution of JH-HLM scores and extended it into the new AMEXO scale using additional response categories. These additional response categories had to present an incremental increase in mobilization, taking highest possible JH-HLM score as the starting point and a walking distance of approximately 1 km as the ceiling. The goal of 1 km after gastrointestinal and oncological surgery was based on our clinical observations and our previous experience of what is achievable after gastrointestinal and oncological surgery [24]. Walking seemed the most appropriate activity to increase mobilization given the context. Other conditions that the new response categories had to meet were that they should be easy to understand for patients and could be easily assessed by healthcare professionals. In between team discussions the additional response categories were pilot tested by a varying composition of nurses, physical therapists and patients to ensure clarity, face validity, and ease of use.

In summary, the AMEXO scale is an extended version of the JH-HLM scale, in which four additional categories (category 9–12) have been added on top of the already existing eight ordinal response categories. Each of the four additional categories presents an incremental increase in mobilization using the highest possible JH-HLM score (i.e., 8 = 250 ft) as a starting point (Additional file 1). This resulted in the following response categories: 9 = 750 ft/225 m (i.e., + 2 times highest possible JH-HLM), 10 = 1500 ft/450 m (i.e., + 3 times highest possible JH-HLM), 11 = 2500 ft/750 m (i.e., + 4 times highest possible JH-HLM) and 12 = 3750 ft/1125 m (i.e., + 5 times highest possible JH-HLM). Using this incremental approach, only four additional response categories were needed to achieve the distance of at least 1 km. Also, each response category could be calculated back to JH-HLM score = 8, providing patients and healthcare professionals with a reference standard to determine the achieved AMEXO score and set mobilization goals together. The AMEXO scale was implemented at the start of February 2019 to replace the JH-HLM in facilitating healthcare professionals in assessing mobilization, setting mobilization goals and discussing mobilization success during inter-professional meetings.

### Ceiling effect

Based on previous research, a ceiling effect was considered to be present if more than 15% of patients achieved the highest possible score [25]. The presence of a ceiling effect when using the JH-HLM and AMEXO scales to assess mobilization was therefore determined by evaluating the percentage of patients with the highest possible mobility score on the first postoperative day before (i.e., JH-HLM score = 8) and after extending the JH-HLM into the AMEXO scale (i.e., AMEXO score = 12). Moreover, the presence of a ceiling effect was also determined by evaluating the percentage of patients with the highest possible mobility score on each of the first three postoperative days. Because a ceiling effect might also affect the responsiveness of the measurement tool[25], the percentage of patients who showed a change in mobility score during the first three postoperative days before and after was evaluated. A change was defined as a difference in mobility score of at least one point during one of the first three postoperative days. Whether this may have been related to the four additional response categories 9 to 12 was evaluated by assessing the number of patients that showed both a change in mobility score and scored 9 to 12 using the AMEXO scale during one of the first three postoperative days.

### Procedures

Routine care data registration procedures before and after were the same. All mobility scores were based on a patient’s mobilization (i.e., what a patient has actually done) over a fixed observation period (e.g. nurse shifts or physical therapist session) [13]. Nurses were instructed to document the mobilization, at the end of each day and evening shift, using the mobility scale implemented at that time. The highest level of mobilization that the nurse observed during her shift was documented in the patient’s electronic medical record and used to set mobilization goals and discuss mobilization success inter-professionally and with the patient [17]. All patients who were admitted to one of the surgical wards received a leaflet with information about the JH-HLM scale or AMEXO scale. Additionally, patients were informed by the nurses on the use of mobility scale and were asked to keep track of their mobility scores in addition to the health care professionals. In correspondence with the study performed by Hoyer et al. [12], the JH-HLM and AMEXO scale were used by nurses, physicians, and physical therapists to discuss mobilization success, barriers to mobilizing patients, set mobilization goals and facilitate discharge planning in routine clinical care.

The following patient characteristics were collected: age, sex, surgical area, acute admission and hospital length of stay. Furthermore, the Katz Activities of Daily Living (Katz-ADL) score[26] and the John Hopkins Fall Risk Assessment Tool (JHFRAT)[27] were collected and used to provide insight into the independence in physical functioning.

### Data analysis

All analysis were conducted using IBM-SPSS Statistics version 26 (IBM Corp, Armonk, New York) and R (R core team, Vienna, Austria). A two-tailed p-value of 0.05 was considered statistically significant. Normality of data was evaluated by visually inspecting continuous and ordinal data using Q-Q plots. Patient characteristics were described descriptively and differences in patient characteristics before-after extending the JH-HLM into the AMEXO scale were assessed using independent *t*-tests, Mann–Whitney U tests or Fisher’s Exact tests, depending on normality and type of data. Due to low number of patients having Katz-ADL score 1 to 6, we recoded this variable to a binary variable (i.e., number of patients scoring 1 to 6 vs number of patients scoring 0).

Due to the fact that > 15% of the mobility scores on the second and third postoperative days were missing a multiple-imputation model with 10 imputed sets was applied to both variables and pooled using Rubin’s rules [28, 29]. Missing data were imputed using all patient characteristics, the mobility score of the first postoperative day, and if available, mobility scores of the second or third postoperative day. Because of the non-normal distribution of missing data predictive mean matching was used [28]. Evaluation of the ceiling effect was performed on both the dataset before imputation as well as after imputation; results were presented separately.

Only the highest mobility score on each postoperative day was used for analysis, as has been in previous studies using the JH-HLM scale to assess mobilization [17]. First, the percentage of patients scoring the highest possible mobility score on the first three postoperative days and the percentage of patients who showed a change in mobility score during the first three postoperative days were analyzed descriptively. Univariable and multivariable logistic regression analyses with backward selection were used to assess the before-after differences with respect to (1) the percentage of patients scoring the highest possible mobility score on the first postoperative day, (2) the percentage of patients scoring the highest possible mobility score during one of the first three postoperative days, and (3) the percentage of patients who showed a change in mobility score during the first three postoperative days. Odds-ratio’s (ORs) and their associated 95% confidence intervals (CIs) were calculated to describe the before-after differences. Patient characteristics that differed significantly before-after extending the JH-HLM into the AMEXO scale were considered as covariates.

## Results

### Study population

**Fig. 1:**
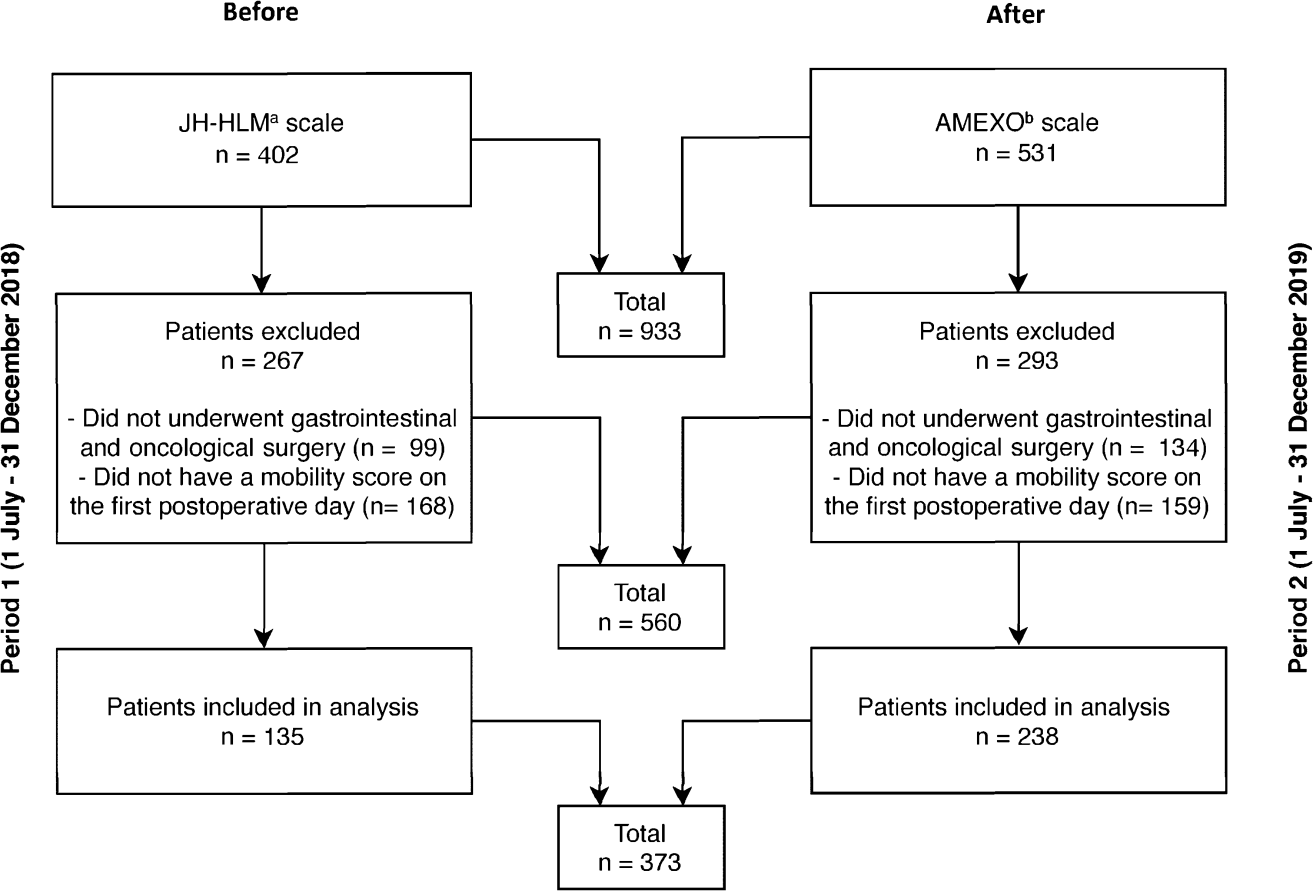
Flow diagram of patient inclusion. ^a^John Hopkins Highest Level of Mobility; ^b^AMsterdam UMC Extension of the John Hopkins Highest Level of mObility

Overall, 933 surgical patients were assessed for eligibility (before n = 402; after n = 531), of whom 560 were excluded (60%). Main reasons for exclusion were no gastrointestinal and oncological surgery (before n = 99; after n = 134) or no mobility score on the first postoperative day (before n = 168; after n = 159). Consequently, a total of 373 patients (before n = 135; after n = 238) were included for analysis (Fig. 1).

Of the 135 patients whose mobilization was assessed before extending the JH-HLM into the AMEXO scale, 65 (48.0%) patients had a JH-HLM mobility score on each of the first three postoperative days. In 35 (25.9%) patients the JH-HLM mobility score was missing on the second postoperative day, and in 60 (44.4%) patients the JH-HLM mobility score was missing on the third postoperative day. Of the 238 patients whose mobilization was assessed after extending the JH-HLM into the AMEXO scale, 73 (30.7%) patients had a AMEXO mobility score on each of the first three postoperative days. In 99 (41.6%) patients the AMEXO mobility score was missing on the second postoperative day, and in 134 (56.3%) patients the AMEXO mobility score was missing on the third postoperative day. The distribution of mobility scores on all three postoperative days before and after extending the JH-HLM into the AMEXO scale can be found in Additional file 2 (before imputation) and Table 1 (after imputation).

**Table 1:** Distribution of the JH-HLM and AMEXO mobility scores during the first three postoperative days (after imputation data)

Measurement instrument		JH-HLM^a^ scale (n = 135)			AMEXO^b^ scale (n = 238)		
Postoperative day, mobility score^c^ (n, %)		Day 1	Day 2	Day 3	Day 1	Day 2	Day 3
Score 12	Walking approximately 3750 ft / 1125 m or more	N/A	N/A	N/A	4 (1.7)	18 (7.6)	26 (10.9)
Score 11	Walking approximately 2500 ft / 750 m or more	N/A	N/A	N/A	9 (3.8)	7 (2.9)	25 (10.5)
Score 10	Walking approximately 1500 ft / 450 m or more	N/A	N/A	N/A	17 (7.1)	20 (8.4)	28 (11.8)
Score 9	Walking approximately 750 ft / 225 m or more	N/A	N/A	N/A	28 (11.8)	64 (26.9)	53 (22.3)
Score 8	Walking approximately 250 ft / 75 m or more	61 (45.2)	98 (72.6)	94 (69.6)	73 (30.7)	71 (29.8)	57 (24.0)
Score 7	Walking approximately 25 ft / 7.5 m or more	17 (12.6)	12 (8.9)	21 (15.6)	38 (16.0)	32 (13.4)	27 (11.3)
Score 6	Walking 10 or more steps	16 (11.9)	9 (6.7)	9 (6.7)	11 (4.6)	8 (3.4)	4 (1.7)
Score 5	Standing for greater than or equal to 1 min	6 (4.4)	3 (2.2)	7 (5.2)	16 (6.7)	6 (2.5)	7 (2.9)
Score 4	Transferring to chair	20 (14.8)	8 (5.9)	3 (2.2)	16 (6.7)	5 (2.1)	5 (2.1)
Score 3	Sitting at edge of bed	11 (8.2)	4 (3.0)	1 (0.7)	12 (5.0)	3 (1.3)	3 (1.3)
Score 2	Bed activities	3 (2.2)	1 (0.7)	0 (0.0)	6 (2.5)	4 (1.7)	3 (1.3)
Score 1	Only lying	1 (0.7)	0 (0.0)	0 (0.0)	8 (3.4)	0 (0.0)	0 (0.0)

*ft.* feet, *m.* meters, *N/A* not applicable

^a^John Hopkins Highest Level of Mobility; ^b^Amsterdam UMC Extension of the John Hopkins Highest Level of mObility; ^c^the highest mobility score achieved on each postoperative day (i.e., 24 h), assessed per nursing shift (e.g., day shift with ambulation distance of 300 m and evening shift with ambulation distance of 460 m means AMEXO 10 on that postoperative day)

### Patient characteristics

Patient characteristics are presented in Table 2. No significant differences before-after extending the JH-HLM into the AMEXO scale were observed for all patient characteristics (*p* > 0.05), except for the number of elective hospital admissions (*p* = 0.024).

**Table 2:** Patient characteristics

Characteristics	JH-HLM^a^ scale (n = 135)	AMEXO^b^ scale (n = 238)	*p* values
Age (years) (median, IQR)	63 (50–71)	64 (51–72)	*p* = 0.647
Sex (male) (n, %)	72 (53.3)	149 (62.6)	*p* = 0.100
Surgical area (n, %)			*p* = 0.827
Upper gastrointestinal surgery	29 (21.5)	56 (23.5)	
Hepato-pancreato-biliary surgery	46 (34.1)	84 (35.3)	
Colorectal surgery	60 (44.4)	98 (41.2)	
Number of elective admissions (n, %)	129 (95.6)	211 (88.7)	*p* = 0.024
Hospital length of stay (days) (median, IQR)	7 (5–11)	7 (5–12)	*p* = 0.616
Missing data (n, %)*	0 (0.0)	1 (0.4)	
Katz activities of daily living score^c^ (n, % of patients scoring ≥ 1)	1 (2.2)	5 (6.2)	*p* = 0.416
Missing data (n, %)*	89 (65.9)	157 (66.0)	
John Hopkins fall risk assessment tool			*p* > 0.999
Low risk (n, %)	46 (34.1)	81 (34.0)	
Moderate risk (n, %)	0 (0.0)	0 (0.0)	
High risk (n, %)	0 (0.0)	0 (0.0)	
Missing (n, %)*	89 (65.9)	157 (66.0)	

^a^John Hopkins Highest Level of Mobility; ^b^Amsterdam UMC Extension of the John Hopkins Highest Level of mObility; *Missing data only reported if present; ^c^number of patients scoring 1 to 6 on the Katz Activities of Daily Living

### Ceiling effect

Sixty-one of the 135 (45.2%) patients scored the highest possible mobility score on the first postoperative day before extending the JH-HLM into the AMEXO scale (i.e., JH-HLM = 8). When divided into subgroups, 20/29 (68.9%) patients after upper gastrointestinal surgery, 17/46 (36.9%) patients after hepato-pancreato-biliary surgery, and 24/60 (40%) patients after colorectal surgery scored the highest possible mobility score on the first operative day before extending the JH-HLM into the AMEXO scale. In contrast, 4/238 (1.7%) patients scored the highest possible mobility score on the first postoperative day after extending the JH-HLM into the AMEXO scale (i.e., AMEXO = 12) (OR = 0.021, CI = 0.007–0.059, *p* < 0.001). Furthermore, 118/135 (87.4%) patients scored the highest possible mobility score on one of the first three postoperative days before, compared to 40/238 (16.8%) patients after extending the JH-HLM into the AMEXO scale (OR = 0.028, CI = 0.013–0.060, *p* < 0.001). A change in mobility score was observed in 88/135 (65.2%) patients before, compared to 225/238 (94.5%) patients after extending the JH-HLM into the AMEXO scale (OR = 9.101, CI = 4.046–20.476, *p* < 0.001). Of these 225 patients, 165 (73.3%) patients showed a change in mobility score and scored AMEXO scale response category 9 to 12 on one of the first three postoperative days. The number of elective hospital admissions did not significantly affect the logistic regression models. A complete case analysis using the before imputation dataset can be found in Additional file 3.

## Discussion

This study demonstrated that healthcare professionals frequently experienced a ceiling effect when they use the JH-HLM scale to assess mobilization after gastrointestinal and oncological surgery. In 87.4% of the patients, the highest possible mobility score was used at least once during the first three postoperative days. And in almost half of the patients, the highest possible mobility score was already used on the first postoperative day. Extending the JH-HLM by adding four additional response categories into the AMEXO scale resulted in a significant decrease of this ceiling effect. Moreover, a change in mobility score was more frequently observed and in 69.2% of the patients this change in mobility score was combined with the use of response category 9 to 12.

Previous studies did not report a ceiling effect when healthcare professionals use the JH-HLM scale to assess mobilization in adult patients admitted to general medicine, neuroscience or general surgical ward [11–13, 17, 20, 21, 30]. The ceiling effect found in this study might be explained by the fact that ceiling effects are often encountered when an existing scale is applied to a new target population [25]. Initially, the JH-HLM scale was developed for a general medicine patient population [12] and the patients previously assessed using the JH-HLM scale were similar to this patient population as many of these patients were acutely admitted with diseases warranting immediate medical attention (e.g., infection diseases, craniotomy, stroke, chronic obstructive pulmonary disease exacerbation). This is in contrast with our study sample of patients admitted for gastrointestinal and oncological surgery. Almost all admissions were planned, which allowed healthcare professionals to screen patients before surgery and, in case it was necessary, offer them the opportunity to follow some form of prehabilitation [31]. This was substantiated by our data on the patient’s fall risk and independence in daily activities—little to none of the patients included in our sample had risk of risk or was limited in activities of daily living before surgery.

In line with previous studies, the majority of patients showed a change in mobility score when healthcare professionals used the JH-HLM to assess mobilization of hospitalized patients [12, 17, 21, 30]. However, our findings also show that when healthcare professionals used the AMEXO scale to assess early postoperative mobilization instead, the number of patients who show a change in mobility score during the first three postoperative days was significantly higher. And in 69.2% of the patients, this change in mobility score was combined with the use of the four newly added response categories 9 to 12. These findings indicate that extending the JH-HLM into the AMEXO scale might not only have reduced the ceiling effect, but might also have improved the scale’s ability to detect mobilization changes during postoperative care [25, 32].

Many different measurement instruments are currently available to assess aspects of ‘mobility’ in hospitalized adult patients, including the de Morton Mobility Index (DEMMI), Hierarchical Assessment of Balance and Mobility (HABAM), Short Physical Performance Battery (SPPB), Performance Oriented Mobility Assessment (POMA), Elderly Mobility Scale (EMS) and the AM-PAC “6-clicks” Basic Mobility short form [13, 23, 33, 34]. Almost all of these measurement instruments, however, focus on the ‘mobility’ aspect of what the patient is capable of doing in a standardized environment. In previous research, this has often been described as the mobility capacity [14] or motor capacity [35] of the patient. In contrast, the JH-HLM and the AMEXO scale are both measurement tools used to assess what patients actually do in ‘current’ (usual) environment, often referred to as mobility performance [14, 35]. While this is a very relevant distinction, the term mobility is often used interchangeably in both research and clinical practice. The John Hopkins University Hospital solved this by using the AM-PAC "6-clicks" Basic Mobility short form on one side and the JH-HLM on the other [13]. Other tools that can be used alongside the JH-HLM or AMEXO scale to assess the mobility capacity after gastrointestinal and oncological surgery instead could be the DEMMI or the SPPB; however, advantages and disadvantages in terms of validity, reliability, responsiveness in patients after gastrointestinal and oncological surgery as well as applicability and usability in routine clinical care should be considered.

### Strengths and limitations

A strength of this study is the fact that all patients after gastrointestinal and oncological surgery who were admitted to two wards in a university medical center were included. Although the population in non-university hospitals may differ, most hospitals have implemented ERAS program and early mobilization has become universal in surgical practices worldwide [2, 3]. Therefore, the results of this study are most likely generalizable to other similar surgical settings.

This study also has to be interpreted in light of some limitations. First, our nonrandomized, uncontrolled before and after study design does not allow us to conclude a direct causation between extending the JH-HLM into the AMEXO scale and the reduction in ceiling effect [36]. Second, although several patient characteristics were considered in our analysis as covariates, we cannot rule out the possibility that other potentially important covariates we were unable to extract from the hospital administrative system (e.g., co-morbidities, type of surgical procedures) may have influenced the observed differences in ceiling effects. Third, because there is no difference in documentation procedures before and after extending the JH-HLM into the AMEXO scale, we hypothesize there are no substantial differences in underlying reasons for missing data when comparing these two groups. Instead, we believe that the before-after differences in missing data may have been caused by an increased documentation of the first postoperative day (i.e., the inclusion criteria) due to the implementation of the AMEXO scale. Still, certain patient groups (e.g., patients with significant mobility and/or cognitive impairments, patients who mobilize faster than expected) may be underrepresented throughout the entire study, therewith limiting the generalizability of our findings. Fourth, within this study a ceiling effect was defined as scoring the highest possible mobility score. As described by Braun et al., patients who score within the minimal detectable change (MDC) of the highest possible score can also be regarded as being at the ceiling effect, as a real change could cross the ceiling [33]. Although Hoyer et al. [13] reported a MDC_95_ of 0.6 for the JH-HLM, the MDC_95_ might have increased over the 1.0 after extending the JH-HLM into the AMEXO scale. Fifth, the scientific limitations that come with single-item measures—such as the JH-HLM and AMEXO—require healthcare professionals, researchers, and policymakers to carefully consider what they intend to use these scales for [37]. While these scales are easy-to-use tools for patients and healthcare professionals to improve mobilization levels during routine clinical care, other more valid and reliable measures for mobility performance should be used to evaluate new interventions.

### Recommendations for future research

Although local healthcare professionals found the AMEXO scale to be a suitable tool to assess mobilization after gastrointestinal and oncological surgery in current clinical care, further psychometric evaluation is warranted. Regarding the validity of the AMEXO scale we would suggest using another tool measuring mobility performance (e.g., accelerometers, concurrent video recordings) to determine whether the response categories of the AMEXO scale are validly assessed after gastrointestinal and oncological surgery (i.e., construct or criterion validity). Furthermore, the inter-rater reliability, test–retest reliability and the responsiveness of the AMEXO scale in patients after gastrointestinal and oncological surgery should be assessed using established methods as described by COSMIN (COnsensus-based Standards for the selection of health Measurement Instruments) [25, 38].

Furthermore, involving patients in improving and sustaining postoperative mobilization has the potential to impact adherence to the ERAS program which is central to effectiveness [39, 40]. The JH-HLM and AMEXO scale have both been developed set individual patient mobilization goals; however, how healthcare professionals can efficiently involve patients in determining these goals and become motivated to achieve them is still unknown. Behavior change techniques such as goal-setting, self-monitoring, instant feedback and reward, have shown to be promising in involving and motivating patients [4]. More insight is needed on how these scales relate to these behavioral change techniques and what else healthcare professionals may need to sustainably improve early mobilization levels in gastrointestinal and oncological surgery patients (e.g., changes to the hospital environment to provide more meaning to ambulation [41]). Although we believe mobility scales are not the only solution to improve early mobilization levels [42], we dare say it is a good first step towards achieving higher mobilization levels in patients after gastrointestinal and oncological surgery.

Lastly, early mobilization entails the incremental increase in activity ranging from passive range-of-motion exercises to active ambulation, depending on the physical capabilities of the patient, from the first day after surgery to reach predetermined targets using a standardized and structured approach [6, 7, 9]. To provide healthcare professionals and policymakers with guidance on how and with what speed mobilization should be increased, future research should explore different early mobilization protocols in relation to surgical outcome, length of stay and mortality. Given the low administrative burden of the AMEXO scale, the AMEXO scale can be used to assess and document the mobilization levels in such studies.

## Conclusions

Healthcare professionals who use the JH-HLM scale to assess early mobilization in patients after gastrointestinal and oncological surgery were frequently hampered by a ceiling effect. Extending the JH-HLM into the AMEXO scale decreased this ceiling effect significantly, making the AMEXO scale more appropriate to assess early mobilization and set daily mobilization goals after gastrointestinal and oncological surgery. Furthermore, the use of the AMEXO scale in patients after gastrointestinal and oncological surgery may provide healthcare professionals with an opportunity to involve patients in creating a culture of safe and improved postoperative mobilization.

## Supplementary Information

**Additional file 1.** Content of the JH-HLM scale and AMEXO scale.

**Additional file 2.** Distribution of mobility scores using the JH-HLM and AMEXO scales during the first three postoperative days (not imputed data).

**Additional file 3.** Comparing the JH-HLM scale with the AMEXO scale (not imputed data).

## Data Availability

The data that support the findings of this study are available from A.M. Eskes, upon reasonable request.

## Abbreviations

AMEXO: AMsterdam UMC EXtension of the JOhn HOpkins Highest Level of mObility
AM-PAC: Activity measure for post-acute care
CI: Confidence intervals
COSMIN: COnsensus-based Standards for the selection of health Measurement Instruments
DEMMI: De Morton Mobility Index
EMS: Elderly Mobility Scale
ERAS: Enhanced recovery after surgery
JH-FRAT: John Hopkins Fall Risk Assessment Tool
JH-HLM: John Hopkins Highest Level of Mobility
HABAM: Hierarchical assessment of balance and mobility
Katz-ADL: Katz Activity of Daily Living
MDC_95_: Minimal Detectable Change_95_
OR: Odds ratio
POMA: Performance Oriented Mobility Assessment
SPPB: Short physical performance battery
STROBE: Strengthening the Reporting of Observational Studies in Epidemiology
