# Supplementary material for: Extended mobility scale (AMEXO) for assessing mobilization and setting goals after gastrointestinal and oncological surgery: a before-after study
Source: BMC Surg. 2022 Feb 2;22:38. doi: 10.1186/s12893-021-01445-3 (PMC8812167; doi:10.1186/s12893-021-01445-3)
Supplement: Supplementary file 1 — Additional file 1. Content of the JH-HLM scale and AMEXO scale. [file 12893_2021_1445_MOESM1_ESM.pdf]

Additional File 1. Content of the JH-HLM[a] scale and AMEXO[b] scale

Mobility scale		Mobility score	Actual mobilization[c]
	AMEXO	**12**	Walking approximately 3750 ft / 1125 m or more
	AMEXO	**11**	Walking approximately 2500 ft / 750 m or more
	AMEXO	**10**	Walking approximately 1500 ft / 450 m or more
	AMEXO	**9**	Walking approximately 750 ft / 225 m or more
JH-HLM	AMEXO	**8**	Walking approximately 250 ft / 75 m or more
JH-HLM	AMEXO	**7**	Walking approximately 25 ft / 7.5 m or more
JH-HLM	AMEXO	**6**	Walking 10 or more steps
JH-HLM	AMEXO	**5**	Standing for greater than or equal to 1 minute
JH-HLM	AMEXO	**4**	Transferring to chair
JH-HLM	AMEXO	**3**	Sitting at edge of bed
JH-HLM	AMEXO	**2**	Bed activities
JH-HLM	AMEXO	**1**	Only lying in bed

Legend: [a] = John Hopkins Highest Level of Mobility; [b] = Amsterdam UMC Extension of the John Hopkins Highest Level of mObility; [c] = on each postoperative day (i.e., 24h), assessed per nursing shift (e.g., day shift with ambulation distance of 300m and evening shift with ambulation distance of 460m means AMEXO 10 on that postoperative day); ft = feet; m = meters
