# Supplementary material for: Extended mobility scale (AMEXO) for assessing mobilization and setting goals after gastrointestinal and oncological surgery: a before-after study
Source: BMC Surg. 2022 Feb 2;22:38. doi: 10.1186/s12893-021-01445-3 (PMC8812167; doi:10.1186/s12893-021-01445-3)
Supplement: Supplementary file 2 — Additional file 2. Distribution of mobility scores using the JH-HLM and AMEXO scales during the first three postoperative days (not imputed data). [file 12893_2021_1445_MOESM2_ESM.pdf]

Additional File 2. Distribution of mobility scores using the JH-HLM and AMEXO scales during the first three postoperative days (not imputed data)

**Measurement instrument**		**JH-HLM[a] scale (n = 135)**			**AMEXO[b] scale (n = 238)**		
Postoperative Day, **Mobility score[c] (n,%)**<br>Missing Data (n)		Day 1	Day 2<br>(n = 35, 25.9%)	Day 3<br>(n = 60, 44.4%)	Day 1	Day 2<br>(n = 99, 41.6%)	Day 3<br>(n = 134, 56.3%)
Score 12	Walking approximately 3750 ft / 1125 m or more	N/A	N/A	N/A	4 (1.7)	9 (3.8)	10 (4.2)
Score 11	Walking approximately 2500 ft / 750 m or more	N/A	N/A	N/A	9 (3.8)	3 (1.3)	9 (3.8)
Score 10	Walking approximately 1500 ft / 450 m or more	N/A	N/A	N/A	17 (7.1)	12 (5.0)	12 (5.0)
Score 9	Walking approximately 750 ft / 225 m or more	N/A	N/A	N/A	28 (11.8)	37 (15.5)	24 (10.1)
Score 8	Walking approximately 250 ft / 75 m or more	61 (45.2)	72 (53.3)	52 (38.5)	73 (30.7)	41 (17.2)	26 (10.9)
Score 7	Walking approximately 25 ft / 7.5 m or more	17 (12.6)	9 (6.7)	12 (8.9)	38 (16.0)	20 (8.4)	12 (5.0)
Score 6	Walking 10 or more steps	16 (11.9)	7 (5.2)	5 (3.7)	11 (4.6)	5 (2.1)	2 (0.8)
Score 5	Standing for greater than or equal to 1 minute	6 (4.4)	2 (1.5)	3 (2.2)	16 (6.7)	4 (1.7)	4 (1.7)
Score 4	Transferring to chair	20 (14.8)	6 (4.4)	2 (1.5)	16 (6.7)	4 (1.7)	2 (0.8)
Score 3	Sitting at edge of bed	11 (8.2)	3 (2.2)	1 (0.7)	12 (5.0)	2 (0.8)	2 (0.8)
Score 2	Bed activities	3 (2.2)	1 (0.7)	0 (0.0)	6 (2.5)	2 (0.8)	1 (0.4)
Score 1	Only lying	1 (0.7)	0 (0.0)	0 (0.0)	8 (3.4)	0 (0.0)	0 (0.0)

Legend: [a] = John Hopkins Highest Level of Mobility; [b] = Amsterdam UMC Extension of the John Hopkins Highest Level of mObility; [c] = the highest mobility score achieved on each postoperative day (i.e., 24h), assessed per nursing shift (e.g., day shift with ambulation distance of 300m and evening shift with ambulation distance of 460m means AMEXO 10 on that postoperative day); ft = feet; m = meters; N/A = not applicable.
