# Supplementary material for: Extended mobility scale (AMEXO) for assessing mobilization and setting goals after gastrointestinal and oncological surgery: a before-after study
Source: BMC Surg. 2022 Feb 2;22:38. doi: 10.1186/s12893-021-01445-3 (PMC8812167; doi:10.1186/s12893-021-01445-3)
Supplement: Supplementary file 3 — Additional file 3. Comparing the JH-HLM scale with the AMEXO scale (not imputed data). [file 12893_2021_1445_MOESM3_ESM.pdf]

Additional File 3. Comparing the JH-HLM scale with the AMEXO scale (not imputed data)

		JH-HLM[a] scale (n = 135)	AMEXO[b] scale (n = 238)	Odds ratio (95% CI)	p-value
Patients achieved the highest mobility score on the first postoperative day[c]	Yes (n,%)<br>No (n,%)	61 (45.2)<br>74 (54.8)	4 (1.7)<br>234 (98.3)	0.021 (0.007-0.059)	< 0.001
Patients achieved the highest mobility score during one of the first three postoperative days[c]	Yes (n,%)<br>No (n,%)	102 (75.6)<br>33 (24.4)	19 (8.0)<br>219 (19.0)	0.028 (0.015-0.052)	< 0.001
Patients showing a change in mobility score[d]	Yes (n,%)<br>No (n,%)<br>Missing data[e]	60 (74.1)<br>21 (25.9)<br>*n* = 54	137 (95.8)<br>6 (4.2)<br>*n* = 95	7.992 (3.070-20.803)	< 0.001
Patients who showed a change in mobility score and scored 9 – 12	Yes (n,%)<br>No (n,%)<br>Missing data[e]	N/A	84 (56.4)<br>65 (43.6)<br>*n* = 89	N/A	N/A

Legend: [a] = John Hopkins Highest Level of Mobility; [b] = Amsterdam UMC Extension of the John Hopkins Highest Level of mObility; [c] = i.e. JH-HLM scale maximum score of 8; AMEXO scale maximum score of 12; [d] = a change is defined as a difference in mobility score of at least one point on one of the first three postoperative days; [e] = unable to compute variable due to missing data on both the second postoperative day and third postoperative day; CI = Confidence intervals; N/A = not applicable.
